# Gait patterns associated with thyroid function: The Rotterdam Study

**DOI:** 10.1038/srep38912

**Published:** 2016-12-14

**Authors:** Arjola Bano, Layal Chaker, Sirwan K. L. Darweesh, Tim I. M. Korevaar, Francesco U. S. Mattace-Raso, Abbas Dehghan, Oscar H. Franco, Jos N. van der Geest, M. Arfan Ikram, Robin P. Peeters

**Affiliations:** 1 Department of Internal Medicine, Erasmus University Medical Center, Rotterdam, The Netherlands; 2 Rotterdam Thyroid Center, Erasmus University Medical Center, Rotterdam, The Netherlands; 3 Department of Epidemiology, Erasmus University Medical Center, Rotterdam, The Netherlands; 4 Department of Epidemiology, Harvard T.H. Chan School of Public Health, Boston, Massachusetts, USA; 5 Section of Geriatric Medicine, Erasmus University Medical Center, Rotterdam, The Netherlands; 6 Department of Neuroscience, Erasmus University Medical Center, Rotterdam, The Netherlands; 7 Department of Neurology, Erasmus University Medical Center Rotterdam, Rotterdam, The Netherlands; 8 Department of Radiology, Erasmus University Medical Center, Rotterdam, The Netherlands

## Abstract

Gait is an important health indicator and poor gait is strongly associated with disability and risk of falls. Thyroid dysfunction is suggested as a potential determinant of gait deterioration, but this has not been explored in a population-based study. We therefore investigated the association of thyroid function with gait patterns in 2645 participants from the Rotterdam Study with data available on TSH (thyroid-stimulating hormone), FT4 (free thyroxine) and gait, without known thyroid disease or dementia. The primary outcome was Global gait (standardized Z-score), while secondary outcomes included gait domains (Rhythm, Variability, Phases, Pace, Base of support, Tandem, Turning) and velocity. Gait was assessed by electronic walkway. Multivariable regression models revealed an inverted U-shaped association of TSH (p < 0.001), but no association of FT4 concentrations with Global gait (p = 0.2). TSH levels were positively associated with Base of support (p = 0.01) and followed an inverted U-shaped curve with Tandem (p = 0.002) and velocity (p = 0.02). Clinical and subclinical hypothyroidism were associated with worse Global gait than euthyroidism (β = −0.61; CI = −1.03, −0.18; p = 0.004 and β = −0.13; CI = −0.26, −0.00; p = 0.04, respectively). In euthyroid participants, higher thyroid function was associated with worse gait patterns. In conclusion, both low and high thyroid function are associated with alterations in Global gait, Tandem, Base of support and velocity.

Gait is an important marker of general health. Disturbances in gait gradually increase with advancing age and affect approximately one third of community-dwelling individuals older than 60 years^1^. Gait impairment has a substantial impact on quality of life and is strongly associated with increased risk of falls, which can in turn cause soft-tissue injuries, fractures and death^2^,^3^. Quantitative gait assessment comprises many parameters that can be summarized into seven independent domains, namely Rhythm, Variability, Phases, Pace, Base of support, Tandem and Turning (Fig. 1)^4^,^5^. These gait domains reflect distinct functional abilities and their investigation is crucial to identify novel modifiable contributors to gait deterioration^5^.

Thyroid hormones regulate metabolism in most tissues, including neurological and musculoskeletal systems, whose integrated functioning is reflected in gait^6^,^7^,^8^. As gait disturbances, thyroid dysfunction increases in prevalence with advancing age. However, the clinical symptoms of thyroid dysfunction become less pronounced among older adults^9^ and this may result in a diagnostic delay and increased risk of systemic complications. Research to date has suggested a possible role of thyroid dysfunction in gait impairment. Adult mice lacking the thyroid-hormone activating enzyme type 2 deiodinase have shown progressive gait impairment in the late stages of life^10^. In humans, several case series^11^,^12^ and case reports^13^,^14^,^15^ have shown a restoration of gait disturbances after treatment of thyroid disease.

Thyroid function in the general population has been linked to gait velocity, which constitutes only one of the parameters in the Pace domain^16^,^17^. However, the link of thyroid function with gait and its spatiotemporal aspects remains unexplored. Therefore, we aimed to investigate the association of thyroid function with Global gait and its separate domains, in a large population-based cohort of middle-aged and elderly subjects.

## Results

We included a total of 2645 eligible participants with data available on thyroid function and gait, without known thyroid disease or dementia (Supplementary Figure 1). The baseline characteristics of the study population are shown in Table 1. The mean age was 59.6 years and 52.6% were females (Table 1).

### The association of thyroid function with Global gait

Our results did not change after primary and additional adjustments for potential confounders; therefore we further report only the most adjusted model (Model 2). TSH concentrations within the full range followed an inverted U-shaped curve with respect to Global gait (p-value < 0.001) (Fig. 2a). However, there was no association of FT4 concentrations with Global gait (p = 0.2) (Fig. 2b). When we restricted the analysis to euthyroid participants, higher TSH concentrations were associated with a better Global gait (β, 0.08; 95% confidence interval [CI], 0.02 to 0.13 per 1 unit logTSH; p = 0.006) (Supplementary Table S1). Moreover, there was a borderline statistically significant association between FT4 levels within the normal range and Global gait (β = −0.05; CI = −0.10 to 0.00 per 1 pmol/L FT4; p = 0.05) (Supplementary Table S1). Clinical and subclinical hypothyroidism were associated with a worse Global gait than euthyroidism (β = −0.61; CI = −1.03 to −0.18; p = 0.004 and β, −0.13; CI, −0.26 to −0.00; p = 0.04 respectively) (Fig. 3a). No association was observed between TPOAb and Global gait in the main analysis or after restricting to euthyroid participants (Supplementary Table S2). Results remained similar after excluding participants with prevalent stroke and Parkinson’s disease (Supplementary Figure 2).

### The associations of thyroid function with gait domains and gait velocity

TSH levels were positively linearly associated with Base of support (p = 0.01) (Fig. 4e) and followed an inverted U-shaped curve with respect to Tandem (p = 0.002) (Fig. 4f) and gait velocity (p = 0.02) (Fig. 2c). In euthyroid participants, higher TSH levels were associated with higher Base of support (β = 0.07; CI = 0.01 to 0.14; p = 0.01) and Tandem (β = 0.06; CI = 0.01 to 0.12; p = 0.04), whereas higher FT4 levels were associated with lower gait velocity (β = −0.96; CI = −1.85 to −0.07; p = 0.03) (Supplementary Table S1). Clinical and subclinical hypothyroidism were associated with lower gait velocity than euthyroidism, with borderline statistical significance (β = −7.11; CI = −14.69 to 0.49; p = 0.06 and β = −2.22; CI = −4.50 to 0.05; p = 0.05, respectively) (Fig. 3b). Gait velocity decreased gradually from euthyroidism to clinical hypothyroidism (p-trend 0.01) (Fig. 3b).

## Discussion

In a large cohort of middle-aged and elderly subjects, we reported an inverted U-shaped association between TSH concentrations and Global gait, indicating that both low and high thyroid function are associated with worse gait. TSH levels were positively associated with Base of support and followed an inverted U-shaped curve with Tandem and gait velocity. In euthyroid subjects, higher thyroid function was associated with worse gait patterns.

The association between thyroid function and gait could be explained by different pathophysiological mechanisms, particularly involving the neurological and musculoskeletal systems. Low and high thyroid function may increase the risk of stroke via unfavorable cardiovascular risk profile and atrial fibrillation, respectively^18^,^19^. Low thyroid function can additionally induce immune-mediated cerebellar degeneration^14^. Furthermore, low and high thyroid function can lead to a dysregulation of the neurotransmission systems and subsequent depressive symptoms^20^. Low and high thyroid function may also contribute to myopathy and fractures, by affecting muscle mass and bone mineral density^21^,^22^. In turn, stroke, cerebellar degeneration, depression, myopathy and fractures are all implicated in gait deterioration^14^,^18^,^19^,^20^,^21^,^22^. In our study, adjustments for stroke, cerebellar cortical volume, TPOAb, CESD depression score, hip and knee pain or stiffness (proxy for musculoskeletal dysfunction) did not change the results, suggesting that the association between thyroid function and gait patterns is independent of these factors. Alternative underlying pathways can explain the association. The most plausible may be peripheral neuropathy, since thyroid dysfunction has been commonly associated with axonal degeneration and nerve conduction abnormalities^21^,^23^,^24^. Both hypothyroid and hyperthyroid patients usually experience symmetric distal sensory disturbances that can resolve after treatment of thyroid dysfunction^21^,^25^. Also, genetic disorders affecting thyroid hormone transport and metabolism may play a role in gait impairment^26^. However, the exact mechanisms through which thyroid function could affect the gait patterns remain unexplored and further studies should be directed towards unravelling the underlying pathophysiology.

Although gait is a multidimensional concept, gait assessment in prior comparable studies has been limited to the measurement of gait velocity^16^,^17^. A relatively small study (n = 602) reported an association of high-normal FT4 levels with slower walk^17^. A second study reported a faster walk in individuals with mildly elevated TSH levels (4.5–7.0 mU/L) compared with euthyroid individuals^16^. Our conclusions are in line with the results of the first study, but do not support those of the second study. Most likely, the discrepancy between our results and those of the second study may be attributable to differences in TSH reference ranges and thyroid status definitions. In the second study, participants with TSH levels between 4.5 and 7.0 mIU/L were considered to have mild subclinical hypothyroidism, though they lacked FT4 measurements. Instead, we used both TSH and FT4 measurements to define the thyroid status of our participants. Therefore, our conclusions may add valuable information to the ongoing debate on the effects of untreated or undetected subclinical hypothyroidism. Most importantly, our large population-based cohort study extends the previous literature by addressing for the first time the association of thyroid function with Global gait and gait domains. Our results indicate the importance of comprehensive gait evaluation, as we observe a stronger association of thyroid status with Global gait than with gait velocity.

We were able to identify Tandem, Base of Support and gait velocity as spatiotemporal gait aspects related to thyroid function. Likewise, past case reports have described hypothyroid patients with a “wide-based gait” and tandem walking errors on neurological examination^12^,^13^,^14^,^15^. In addition, adult mice lacking type 2 deiodinase walked slower and with wider base of support than the wild-type mice^10^. Our results confirm these findings in the setting of a general population cohort study. Of note, the identification of thyroid-related gait domains may provide valuable *hints* on the pathways linking thyroid function to gait. Tandem, Base of Support and gait velocity have been associated with distinct brain structures (i.e. prefrontal regions, parietal cortex, pallidum, putamen, and cerebellum), executive functioning and balance, that might be specific targets of thyroid hormone action^4^,^15^,^27^,^28^,^29^,^30^,^31^.

A limitation of our study is its cross-sectional design, which does not enable us to draw conclusions on causality. Though it is more likely that thyroid function affects gait than vice-versa, one could also hypothesize that health problems underlying gait abnormalities may alter thyroid parameters in the setting of non-thyroidal illness syndrome (NTIS). This condition is characterized by low thyroid hormones and normal TSH levels^32^. Instead, we reported a non-linear association between TSH levels and Global gait. Also, NTIS is typical in critically ill patients, whereas the RS consists of community-dwelling adults^32^. Therefore, NTIS is unlikely to be the explanation of our findings. Furthermore, turning and tandem walk lacked repeated measurements, which would have reduced the intra-individual variability. However, we did perform up to eight consecutive recordings of the normal walk and used a well validated instrument for an objective gait evaluation in three walking conditions. Also, the RS does not have data available on serum triiodothyronine levels, which is a limitation for most population-based studies. However, TSH and FT4 concentrations are considered as the most relevant measurements of thyroid function in clinical practice. Moreover, RS includes predominantly Caucasians over 45 years old, which limits the generalizability of our findings to other populations. Lastly, the possibility of residual confounding cannot be excluded, even though we controlled for multiple potential confounders.

In summary, both low and high thyroid function are associated with worse gait patterns. There is an inverted U-shaped association of TSH levels with Global gait, Tandem and gait velocity, as well as a positive association of TSH levels with Base of support. Subjects with clinical and subclinical hypothyroidism have worse gait patterns than euthyroid individuals. These conclusions might have future implications regarding the prevention and treatment of thyroid and gait disorders. Further studies are needed to confirm our findings, determine the underlying mechanisms linking thyroid function to gait patterns and subsequently investigate the possible motor benefits of thyroid treatment.

## Materials and Methods

### Study population

The Rotterdam Study (RS) is an ongoing prospective population-based cohort study that investigates chronic diseases in the middle-aged and elderly. The objectives and study design of RS have been described in detail elsewhere^33^. RS was initiated in 1990, including 7983 participants aged 55 years or older (RS I). In 2000, the cohort was expanded with 3011 participants aged 55 or older (RS II). In 2006, a third cohort of 3932 participants aged 45 years and over was added (RS III). As of now, RS comprises a total of 14926 participants, who undergo extensive follow-up medical examinations every 2 to 4 years. From 2009 onwards, quantitative gait assessment was included in the study protocol. Between March 2009 and March 2012, 3651 participants of the RS were invited for gait assessment. An overview on the selection of study participants can be found in the flowchart (Supplementary Figure 1).

The Medical Ethics Committee of the Erasmus University and the Ministry of Health, Welfare and Sport of the Netherlands have approved the study protocols, implementing the “Wet Bevolkingsonderzoek: ERGO (Population Studies Act: Rotterdam Study)”. The methods were performed in accordance with the approved guidelines. All included participants provided written informed consent in accordance with the Declaration of Helsinki.

### Population for analysis

A total of 2857 subjects had complete information on thyroid function and gait. Of these, we excluded 212 subjects with at least one out of several conditions: 1) dementia diagnosis (n = 14); 2) thyroid medication usage (n = 79); 3) history of thyroid disease (n = 192) and 4) previous thyroid surgery (n = 33) (Supplementary Figure 1). The remaining 2645 eligible participants were enrolled in the study.

### Assessment of thyroid function

Thyroid function tests were performed in study cohorts RS I visit 3 (RS I-3), RS II visit 1 (RS II-1) and RS III visit 1 (RS III.1) using the same method and assay. Concentrations of thyroid-stimulating hormone (TSH), free thyroxine (FT4) and thyroid peroxidase antibodies (TPOAb) were measured on baseline serum samples stored at −80 °C using the electrochemiluminescence immunoassay, “ECLIA”, Roche. We determined the reference range of serum TSH as 0.40–4.0 mIU/L and serum FT_4_ as 11–25 pmol/L (alternatively 0.86–1.94 ng/dL), according to national guidelines and our previous studies^34^,^35^. Euthyroidism was defined as serum TSH within the reference range. Subclinical hypothyroidism was defined as serum TSH > 4.0 mIU/L and FT4 levels within the reference range. Overt hypothyroidism was defined as serum TSH > 4.0 mIU/L and FT4 levels < 11 pmol/L. Subclinical hyperthyroidism was defined as serum TSH < 0.40 mIU/L and FT4 levels within the reference range. Overt hyperthyroidism was defined as serum TSH < 0.40 mIU/L and FT4 levels > 25 pmol/L. TPOAb positivity (reflecting thyroid autoimmunity) was defined as TPOAb levels above the cut-off of 35 kU/ml, in accordance with the recommendations of the assay manufacturer^34^,^35^.

### Assessment of gait

**Figure 1:**
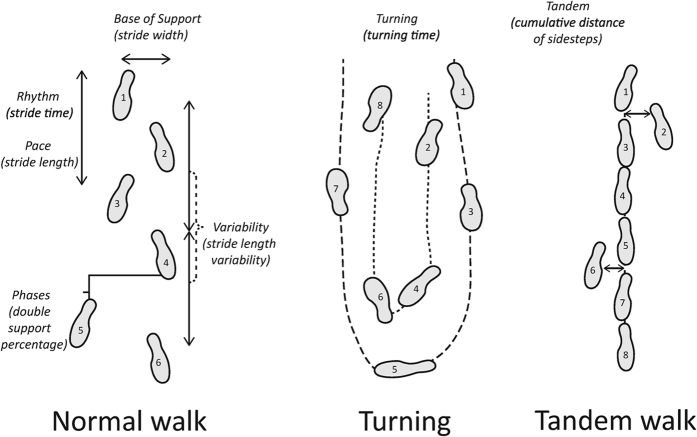
Normal walk, Turning and Tandem. The three walking conditions, including five gait domains for normal walk (Rhythm, Variability, Phases, Pace, Base of support), one for turn (Turning) and one for tandem walk (Tandem).

**Figure 2:**
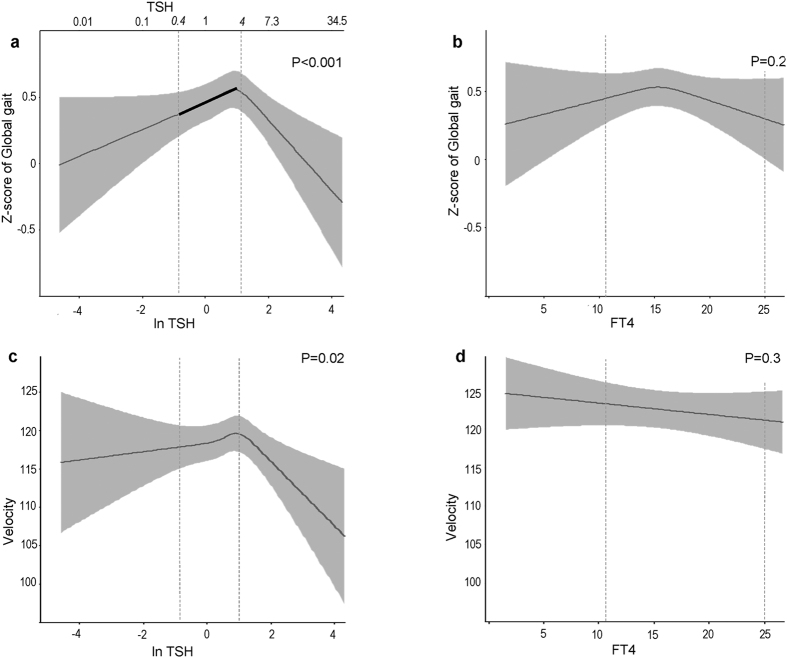
Association of thyroid function with Global gait and velocity. Adjusted for age, sex, cohort, smoking, alcohol intake, education level, height, weight, time interval between thyroid function measurement and gait assessment, knee pain or stiffness, hip pain or stiffness, prevalent stroke, CESD depression score, cerebellar cortical volume, intracranial volume, thyroid peroxidase antibodies. We utilized linear regression models with restricted cubic splines. TSH/FT4 concentrations are plotted against predicted means of Z-score Global gait and velocity (black lines) with 95% CI (gray areas). Dashed lines indicate the limits of TSH or FT4 reference ranges. A higher value of Global gait represents better gait.

**Figure 3:**
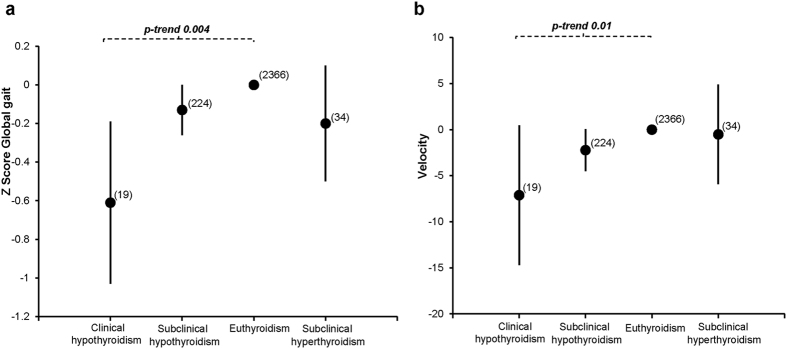
Association of thyroid status categories with Global gait and velocity. Adjusted for age, sex, cohort, smoking, alcohol intake, education level, height, weight, time interval between thyroid function measurement and gait assessment, knee pain or stiffness, hip pain or stiffness, prevalent stroke, CESD depression score, cerebellar cortical volume, intracranial volume, thyroid peroxidase antibodies. Thyroid status categories are plotted against differences in Z score of Global gait and velocity, with euthyroid subjects as reference. Euthyroidism was defined as TSH (thyroid-stimulating hormone) within reference range (0.4–4.0 mIU/l); clinical hypothyroidism as TSH > 4.0 mU/L and FT4 (free thyroxine) <11 pmol/L; subclinical hypothyroidism as TSH > 4.0 mU/L and FT411-25 pmol/L; clinical hyperthyroidism as TSH < 0.4 mU/L and FT4 > 25 pmol/L; subclinical hyperthyroidism as TSH < 0.4 mU/L and FT411-25 pmol/L. None of the participants had clinical hyperthyroidism. Error bars represent the 95% confidence intervals around the standardized β (black dots). Within brackets: Total number. A higher value of global gait represents better gait.

**Figure 4:**
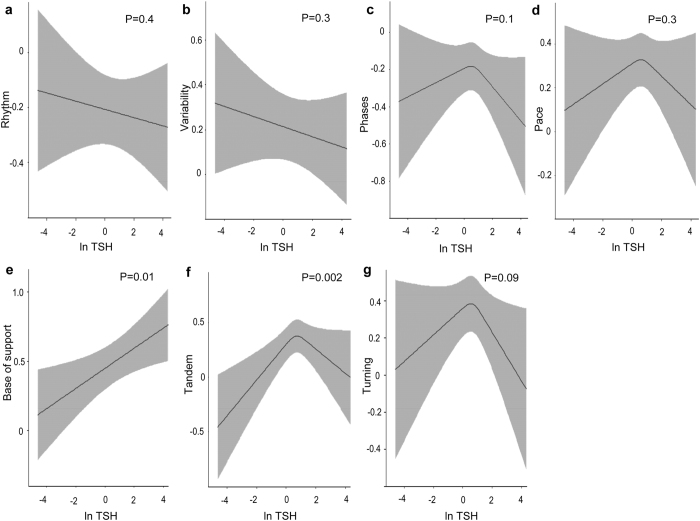
Association of TSH with the seven gait domains. Adjusted for age, sex, cohort, smoking, alcohol intake, education level, height, weight, time interval between thyroid function measurement and gait assessment, knee pain or stiffness, hip pain or stiffness, prevalent stroke, CESD depression score, cerebellar cortical volume, intracranial volume, thyroid peroxidase antibodies. The model including Tandem walk was additionally adjusted for step count and mean step size. Point estimates are reported as predicted means (black lines) of gait domains with 95% CI (gray areas). A higher value of gait domains represents better gait.

Quantitative gait assessment was performed in study cohorts RS I visit 5 (RS I-5), RS II visit 3 (RS II-3) and RS III visit I (RS III.1). Gait was evaluated using a 5.79-m long walkway (GAITRite Platinum; CIR systems, Sparta, NJ: 4.88-m active area; 120-Hz sampling rate). The reliability and validity of this device have been previously established^4^,^36^,^37^,^38^. The standardized gait protocol comprises three walking conditions: normal walk, turning and tandem walk (Fig. 1). In the normal walk, participants walked at their usual pace across the walkway. This walk was repeated eight times, of which the first recording was considered a practice walk and excluded from the analyses. In turning, participants walked at their usual pace, turned halfway, and returned to the starting position. In the tandem walk, participants walked heel-to-toe on a line across the walkway. Based on the recorded footfalls, the walkway software calculated thirty gait parameters, including twenty five from the normal walk, two from turning and three from the tandem walk. Subsequently, principal component analysis (PCA) was performed to avoid multiple testing and collinearity across the variables. While capturing the largest amount of variance, PCA summarizes gait parameters into seven independent gait domains: Rhythm, Variability, Phases, Pace, Base of Support, Tandem and Turning^5^. Rhythm reflects cadence and stride time; Variability reflects variations in length and time among strides; Phases reflects double support time and double support as a percentage of the gait cycle; Pace reflects stride length and gait velocity; Base of Support reflects stride width and stride width variability; Tandem reflects errors in tandem walking; Turning reflects turning time and the number of turn steps^5^. When necessary, gait domains were inverted so that lower values represent “worse” gait. Global gait was calculated by averaging gait domains into a standardized Z-score^5^. Gait velocity was additionally included in our analysis in order to compare our findings with previous studies investigating the association between thyroid function and gait velocity^16^,^17^.

### Assessment of covariates

The baseline home interview provided information on medical history, tobacco smoking, alcohol consumption, education level, medication, knee and hip pain or stiffness. Participants were categorized based on their smoking status (current, past and never smokers) and education level (low, intermediate and high). Height and weight were measured during the examinations at the research center. Stroke cases were reviewed and verified by an experienced vascular neurologist using hospital letters, information from practitioners and nursing home physicians. Depressive disorders were evaluated based on the Centre for Epidemiological Studies Depression Scale (CESD) questionnaire. A score above 16 was considered indicative of a depressive disorder^39^. Cerebellar cortical volume and intracranial volume were examined by standardized magnetic resonance imaging (MRI) scanning of the brain^33^.

### Statistical analysis

We investigated the association of thyroid parameters (TSH, FT4 and TPOAb positivity) with Global gait and spatiotemporal gait components, by performing ordinary least-squares linear regression. The primary outcome was Global gait, while secondary outcomes included gait domains (i.e. Rhythm, Variability, Phases, Pace, Base of support, Tandem and Turning) and gait velocity. We fitted restricted cubic splines to allow for potential nonlinearity. Moreover, we evaluated Global gait and gait velocity throughout thyroid function categories, with euthyroid subjects as reference group. Next, we examined the association of thyroid function with gait in euthyroid participants. In addition, we performed a sensitivity analysis excluding participants with prevalent stroke (n = 66) and Parkinson’s disease (n = 3).

All analyses were adjusted for potential confounding by age, sex, cohort, smoking status, alcohol intake (Model 1). As thyroid function measurement preceded the gait assessment, we also adjusted for the time interval between measurements. In Model 2, we additionally adjusted for covariates that could be either confounders or mediators, including education level, height, weight, knee pain or stiffness, hip pain or stiffness, prevalent stroke, CESD depression score, cerebellar cortical volume, intracranial volume, TPOAb concentrations. Step count and mean step size can affect the score of Tandem walk. Therefore, all models including Tandem walk were further adjusted for step count and mean step size.

TSH values were logarithmically transformed, because of its skewed distribution. The assumption of normally distributed residuals was checked and met. All models were tested for effect modification by separately adding product interaction terms of the exposure (TSH or FT4 or TPOAb) with covariates of the multivariable model, but none of the interaction terms were significant. Multiple imputations were performed for covariates with missing data (less than 4.6% for all covariates). A p-value (two-tailed) <0.05 was considered statistically significant. Statistical analyses were conducted using R statistical software (rms-package, R-project, Institute for Statistics and Mathematics, R Core Team, Vienna, Austria, version 3.2.2) and IBM SPSS version 21 (IBM Corp).

## Additional Information

**How to cite this article:** Bano, A. *et al*. Gait patterns associated with thyroid function: The Rotterdam Study. *Sci. Rep.* **6**, 38912; doi: 10.1038/srep38912 (2016).

**Publisher's note:** Springer Nature remains neutral with regard to jurisdictional claims in published maps and institutional affiliations.

## Supplementary Material

Supplementary Information

## Figures and Tables

**Table 1:** Baseline characteristics of 2645 participants.

Characteristics	Mean (sd)*
Age, years	59.6 (6.6)
Female, n (%)	1392 (52.6)
Smoking, n (%)	
current 561 (21.2)	561 (21.2)
past 1242 (47.0)	1242 (47.0)
never 842 (31.8)	842 (31.8)
Alcohol intake >14 drinks/week, n (%)	565 (21.4)
Education level, n (%)	
low	195 (7.4)
intermediate	1821 (68.8)
high	629 (23.7)
Height, cm	170.0 (9.2)
Weight, kg	78.4 (14.1)
Knee pain or stiffness, n (%)	693 (26.2)
Hip pain or stiffness, n (%)	401 (15.2)
Past stroke, n (%)	66 (2.5)
CESD depressive symptoms, n (%)	298 (11.3)
Cerebellar cortical volume, ml	99.3 (10.6)
Intracranial volume, ml	1479.6 (159.6)
TSH, mIU/L, median (IQR)	1.9 (1.3–2.8)
FT4, pmol/L	15.5 (2.1)
TPOAb positive, n (%)	312 (11.8)

*Data are mean (sd), unless otherwise specified.

Abbreviations: sd, standard deviation; CESD, Centre for Epidemiological Studies Depression Scale; TSH, thyroid-stimulating hormone; IQR, interquartile range; FT4, free thyroxine; TPOAb, thyroid peroxidase antibodies (cutoff 35 kU/ml).

## References

[b1] Mahlknecht P. . Prevalence and burden of gait disorders in elderly men and women aged 60–97 years: a population-based study. PLoS One 8, e69627 (2013). 23894511 10.1371/journal.pone.0069627 PMC3722115

[b2] Sudarsky L. Geriatrics: gait disorders in the elderly. N Engl J Med 322, 1441–1446 (1990). 2184358 10.1056/NEJM199005173222007

[b3] Verghese J., Holtzer R., Lipton R. B. & Wang C. Quantitative gait markers and incident fall risk in older adults. J Gerontol A Biol Sci Med Sci 64, 896–901 (2009). 19349593 10.1093/gerona/glp033 PMC2709543

[b4] Verlinden V. J., van der Geest J. N., Hofman A. & Ikram M. A. Cognition and gait show a distinct pattern of association in the general population. Alzheimers Dement 10, 328–335 (2014). 23849591 10.1016/j.jalz.2013.03.009

[b5] Verlinden V. J. . Gait patterns in a community-dwelling population aged 50 years and older. Gait Posture 37, 500–505 (2013). 23018028 10.1016/j.gaitpost.2012.09.005

[b6] van Doorn J., Roelfsema F. & van der Heide D. Concentrations of thyroxine and 3,5,3′-triiodothyronine at 34 different sites in euthyroid rats as determined by an isotopic equilibrium technique. Endocrinology 117, 1201–1208 (1985). 4017962 10.1210/endo-117-3-1201

[b7] Schwartz H. L. & Oppenheimer J. H. Nuclear triiodothyronine receptor sites in brain: probable identity with hepatic receptors and regional distribution. Endocrinology 103, 267–273 (1978). 217638 10.1210/endo-103-1-267

[b8] Salvatore D., Simonides W. S., Dentice M., Zavacki A. M. & Larsen P. R. Thyroid hormones and skeletal muscle–new insights and potential implications. Nat Rev Endocrinol 10, 206–214 (2014). 24322650 10.1038/nrendo.2013.238 PMC4037849

[b9] Vanderpump M. P. . The incidence of thyroid disorders in the community: a twenty-year follow-up of the Whickham Survey. Clin Endocrinol (Oxf) 43, 55–68 (1995). 7641412 10.1111/j.1365-2265.1995.tb01894.x

[b10] Barez-Lopez S. . Abnormal motor phenotype at adult stages in mice lacking type 2 deiodinase. PLoS One 9, e103857 (2014). 25083788 10.1371/journal.pone.0103857 PMC4118963

[b11] Jellinek E. H. & Kelly R. E. Cerebellar syndrome in myxoedema. Lancet (London, England) 2, 225–227 (1960). 10.1016/s0140-6736(60)91424-0 13853110

[b12] Cremer G. M., Goldstein N. P. & Paris J. Myxedema and ataxia. Neurology 19, 37–46 (1969). 4304453 10.1212/wnl.19.1.37

[b13] Edvardsson B. & Persson S. Subclinical hypothyroidism presenting with gait abnormality. Neurologist 16, 115–116 (2010). 20220447 10.1097/NRL.0b013e3181be6fdb

[b14] Sangle S. A., Lohiya R. V., Sharma D. R. & Bote N. Hypothyroidism - gait matters. J Postgrad Med 58, 159 (2012). 22718066 10.4103/0022-3859.97183

[b15] Barnard R. O., Campbell M. J. & McDonald W. I. Pathological findings in a case of hypothyroidism with ataxia. Journal of neurology, neurosurgery, and psychiatry 34, 755–760 (1971). 10.1136/jnnp.34.6.755 PMC1083515 5158793

[b16] Simonsick E. M. . Subclinical hypothyroidism and functional mobility in older adults. Arch Intern Med 169, 2011–2017 (2009). 19933964 10.1001/archinternmed.2009.392 PMC2879334

[b17] Simonsick E. M., Chia C. W., Mammen J. S., Egan J. M. & Ferrucci L. Free Thyroxine and Functional Mobility, Fitness, and Fatigue in Euthyroid Older Men and Women in the Baltimore Longitudinal Study of Aging. J Gerontol A Biol Sci Med Sci 71, 961–967 (2016). 26791089 10.1093/gerona/glv226 PMC4906324

[b18] Squizzato A., Gerdes V. E., Brandjes D. P., Buller H. R. & Stam J. Thyroid diseases and cerebrovascular disease. Stroke 36, 2302–2310 (2005). 16179578 10.1161/01.STR.0000181772.78492.07

[b19] Chaker L. . Subclinical Hypothyroidism and the Risk of Stroke Events and Fatal Stroke: An Individual Participant Data Analysis. J Clin Endocrinol Metab 100, 2181–2191 (2015). 25856213 10.1210/jc.2015-1438 PMC4454799

[b20] Bauer M., Goetz T., Glenn T. & Whybrow P. C. The thyroid-brain interaction in thyroid disorders and mood disorders. J Neuroendocrinol 20, 1101–1114 (2008). 18673409 10.1111/j.1365-2826.2008.01774.x

[b21] Duyff R. F., Van den Bosch J., Laman D. M., van Loon B. J. & Linssen W. H. Neuromuscular findings in thyroid dysfunction: a prospective clinical and electrodiagnostic study. Journal of neurology, neurosurgery, and psychiatry 68, 750–755 (2000). 10.1136/jnnp.68.6.750 PMC1736982 10811699

[b22] Vestergaard P. & Mosekilde L. Fractures in patients with hyperthyroidism and hypothyroidism: a nationwide follow-up study in 16,249 patients. Thyroid 12, 411–419 (2002). 12097203 10.1089/105072502760043503

[b23] Beghi E. . Hypothyroidism and polyneuropathy. Journal of neurology, neurosurgery, and psychiatry 52, 1420–1423 (1989). 10.1136/jnnp.52.12.1420 PMC1031603 2559162

[b24] El-Salem K. & Ammari F. Neurophysiological changes in neurologically asymptomatic hypothyroid patients: a prospective cohort study. Journal of clinical neurophysiology: official publication of the American Electroencephalographic Society 23, 568–572, doi: 10.1097/01.wnp.0000231273.22681.0e (2006). 17143145

[b25] Kececi H. & Degirmenci Y. Hormone replacement therapy in hypothyroidism and nerve conduction study. Neurophysiol Clin 36, 79–83 (2006). 16844546 10.1016/j.neucli.2006.04.001

[b26] Venero C. . Anxiety, memory impairment, and locomotor dysfunction caused by a mutant thyroid hormone receptor alpha1 can be ameliorated by T3 treatment. Genes Dev 19, 2152–2163 (2005). 16131613 10.1101/gad.346105 PMC1221886

[b27] Rosano C., Aizenstein H. J., Studenski S. & Newman A. B. A regions-of-interest volumetric analysis of mobility limitations in community-dwelling older adults. J Gerontol A Biol Sci Med Sci 62, 1048–1055 (2007). 17895446 10.1093/gerona/62.9.1048

[b28] Soumare A., Tavernier B., Alperovitch A., Tzourio C. & Elbaz A. A cross-sectional and longitudinal study of the relationship between walking speed and cognitive function in community-dwelling elderly people. J Gerontol A Biol Sci Med Sci 64, 1058–1065 (2009). 19561146 10.1093/gerona/glp077

[b29] Watson N. L. . Executive function, memory, and gait speed decline in well-functioning older adults. J Gerontol A Biol Sci Med Sci 65, 1093–1100 (2010). 20581339 10.1093/gerona/glq111 PMC2949334

[b30] Rosano C. . Special article: gait measures indicate underlying focal gray matter atrophy in the brain of older adults. J Gerontol A Biol Sci Med Sci 63, 1380–1388 (2008). 19126852 10.1093/gerona/63.12.1380 PMC2648808

[b31] de Laat K. F. . Cortical thickness is associated with gait disturbances in cerebral small vessel disease. Neuroimage 59, 1478–1484 (2012). 21854857 10.1016/j.neuroimage.2011.08.005

[b32] Fliers E., Bianco A. C., Langouche L. & Boelen A. Thyroid function in critically ill patients. Lancet Diabetes Endocrinol 3, 816–825 (2015). 26071885 10.1016/S2213-8587(15)00225-9 PMC4979220

[b33] Hofman A. . The Rotterdam Study: 2016 objectives and design update. Eur J Epidemiol 30, 661–708 (2015). 26386597 10.1007/s10654-015-0082-x PMC4579264

[b34] Heeringa J. . High-normal thyroid function and risk of atrial fibrillation: the Rotterdam study. Arch Intern Med 168, 2219–2224 (2008). 19001198 10.1001/archinte.168.20.2219

[b35] Chaker L. . Thyroid function and age-related macular degeneration: a prospective population-based cohort study–the Rotterdam Study. BMC Med 13, 94 (2015). 25903050 10.1186/s12916-015-0329-0 PMC4407352

[b36] Menz H. B., Latt M. D., Tiedemann A., Mun San Kwan M. & Lord S. R. Reliability of the GAITRite walkway system for the quantification of temporo-spatial parameters of gait in young and older people. Gait Posture 20, 20–25 (2004). 15196515 10.1016/S0966-6362(03)00068-7

[b37] Webster K. E., Wittwer J. E. & Feller J. A. Validity of the GAITRite walkway system for the measurement of averaged and individual step parameters of gait. Gait Posture 22, 317–321 (2005). 16274913 10.1016/j.gaitpost.2004.10.005

[b38] Rao A. K., Quinn L. & Marder K. S. Reliability of spatiotemporal gait outcome measures in Huntington’s disease. Mov Disord 20, 1033–1037 (2005). 15838854 10.1002/mds.20482

[b39] Luijendijk H. J. . Incidence and recurrence of late-life depression. Arch Gen Psychiatry 65, 1394–1401 (2008). 19047526 10.1001/archpsyc.65.12.1394

