# Supplementary material for: Gait patterns associated with thyroid function: The Rotterdam Study
Source: Sci Rep. 2016 Dec 14;6:38912. doi: 10.1038/srep38912 (PMC5155238; doi:10.1038/srep38912)

Gait patterns associated with thyroid function: The Rotterdam Study

Arjola Bano[1,2,3], Layal Chaker[1,2,3,4], Sirwan K. L. Darweesh[3,4], Tim I. M. Korevaar[1,2], Francesco U.S. Mattace-Raso[1,5], Abbas Dehghan[3], Oscar H. Franco[3], Jos N. van der Geest[6], M. Arfan Ikram[3,7,8*], Robin P. Peeters[1,2,3]

[1]Department of Internal Medicine, Erasmus University Medical Center, Rotterdam, The Netherlands

[2]Rotterdam Thyroid Center, Erasmus University Medical Center, Rotterdam, The Netherlands

[3]Department of Epidemiology, Erasmus University Medical Center, Rotterdam, The Netherlands

[4]Department of Epidemiology, Harvard T.H. Chan School of Public Health, Boston, Massachusetts, USA

[5]Section of Geriatric Medicine, Erasmus University Medical Center, Rotterdam, The Netherlands

[6]Department of Neuroscience, Erasmus University Medical Center, Rotterdam, The Netherlands

[7]Department of Neurology, Erasmus University Medical Center Rotterdam, Rotterdam, The Netherlands

[8]Department of Radiology, Erasmus University Medical Center, Rotterdam, The Netherlands

**Corresponding author*

Supplementary Table S1. Association of thyroid function with Global gait, gait domains and gait velocity in euthyroid participants*

	TSH		FT4	
	β (95% CI)	p-value	β (95% CI)	p-value
Global gait	0.08 (0.02; 0.13)	0.006	-0.05 (-0.10; 0.00)	0.05
Rhythm	0.00 (-0.06; 0.06)	0.8	-0.02 (-0.07; 0.03)	0.4
Variability	0.02 (-0.04; 0.08)	0.4	-0.01 (-0.06; 0.04)	0.7
Phases	0.02 (-0.03; 0.08)	0.4	-0.05 (-0.10; 0.00)	0.05
Pace	0.05 (0.00; 0.10)	0.05	-0.02 (-0.07; 0.02)	0.3
Base of support	0.07 (0.01; 0.14)	0.01	0.01 (-0.04; 0.06)	0.6
Tandem†	0.06 (0.01; 0.12)	0.04	-0.03 (-0.08; 0.02)	0.2
Turning	-0.02 (-0.08; 0.04)	0.4	-0.01 (-0.06; 0.05)	0.8
Velocity	0.87 (-0.12; 1.87)	0.08	-0.96 (-1.85;-0.07)	0.03

A higher value of gait represents better gait.
Analyses are adjusted for age, sex, cohort, smoking, alcohol intake, education level, height, weight, time interval between thyroid function measurement and gait assessment, knee pain or stiffness, hip pain or stiffness, prevalent stroke, CESD depression score, cerebellar cortical volume, intracranial volume, thyroid peroxidase antibodies.
*Euthyroidism was defined as TSH within the reference range (0.4-4.0 mIU/l).
†Additionally adjusted for step count and step size within tandem walk.
Abbreviations: TSH, thyroid-stimulating hormone, is per one unit increase of log transformed TSH (mIU/L); FT4, free thyroxine, is per one unit increase of FT4 (pmol/L); β, regression coefficient; CI, confidence interval.

**Supplementary Table S2. Association of TPOAb positivity* with Global gait**	
	β (95% CI)
All participants	-0.05 (-0.16; 0.06)
Euthyroid participants†	-0.06 (-0.19; 0.06)

A higher value of Global gait represents better gait.
Analyses are adjusted for age, sex, cohort, smoking, alcohol intake, education level, height, weight, time interval between thyroid function measurement and gait assessment, knee pain or stiffness, hip pain or stiffness, prevalent stroke, CESD depression score, cerebellar cortical volume, intracranial volume, lnTSH.
*TPOAb >35 kU/ml were regarded as positive.
†Euthyroidism was defined as TSH within the reference range (0.4-4.0 mIU/l).
Abbreviations: TPOAb, thyroid peroxidase antibodies; TSH, thyroid-stimulating hormone.

Supplementary Figure 1. Flow chart for selection of study participants

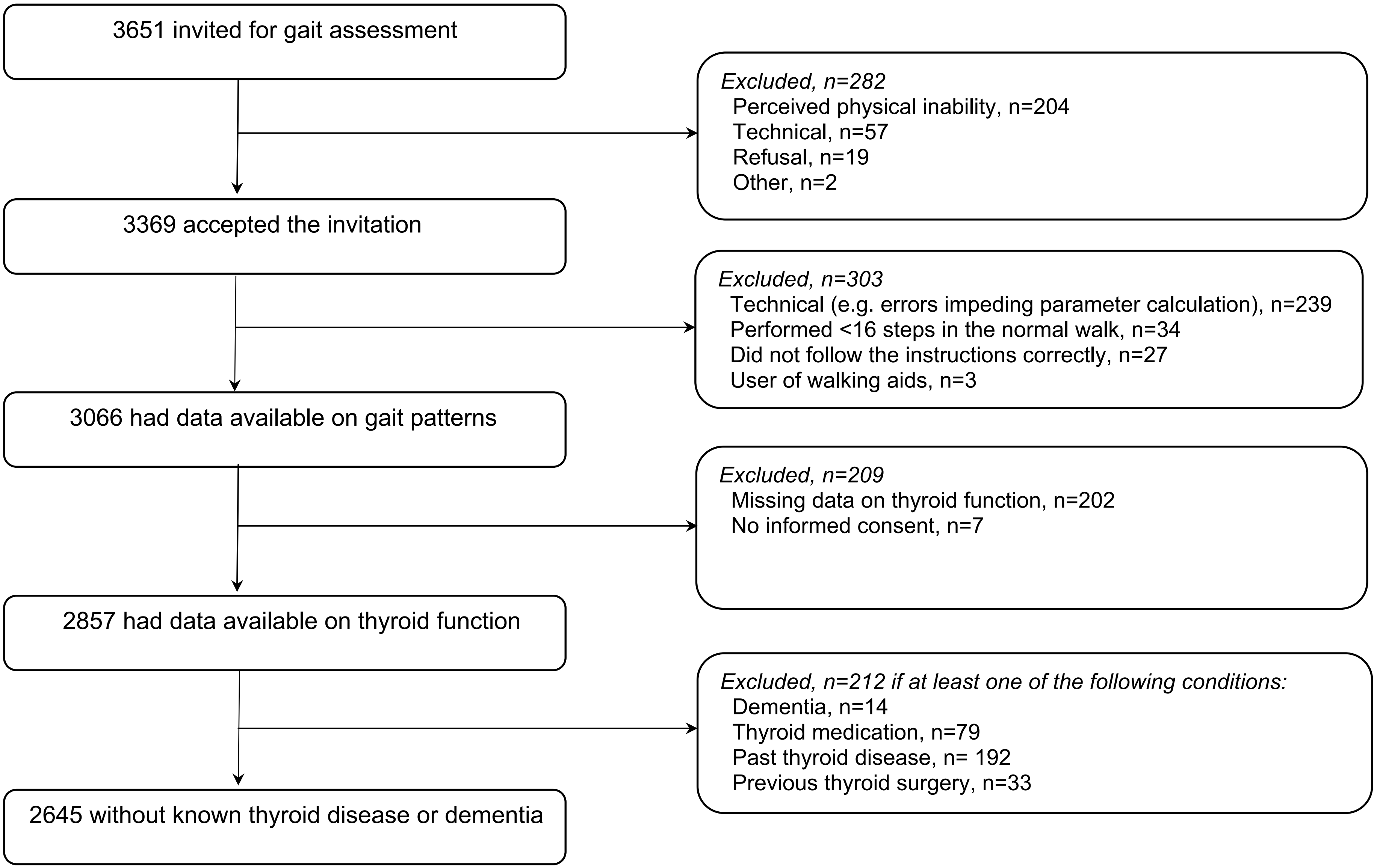

Supplementary Figure 2. Association of thyroid function with Global gait, after excluding participants with prevalent stroke and Parkinson's disease

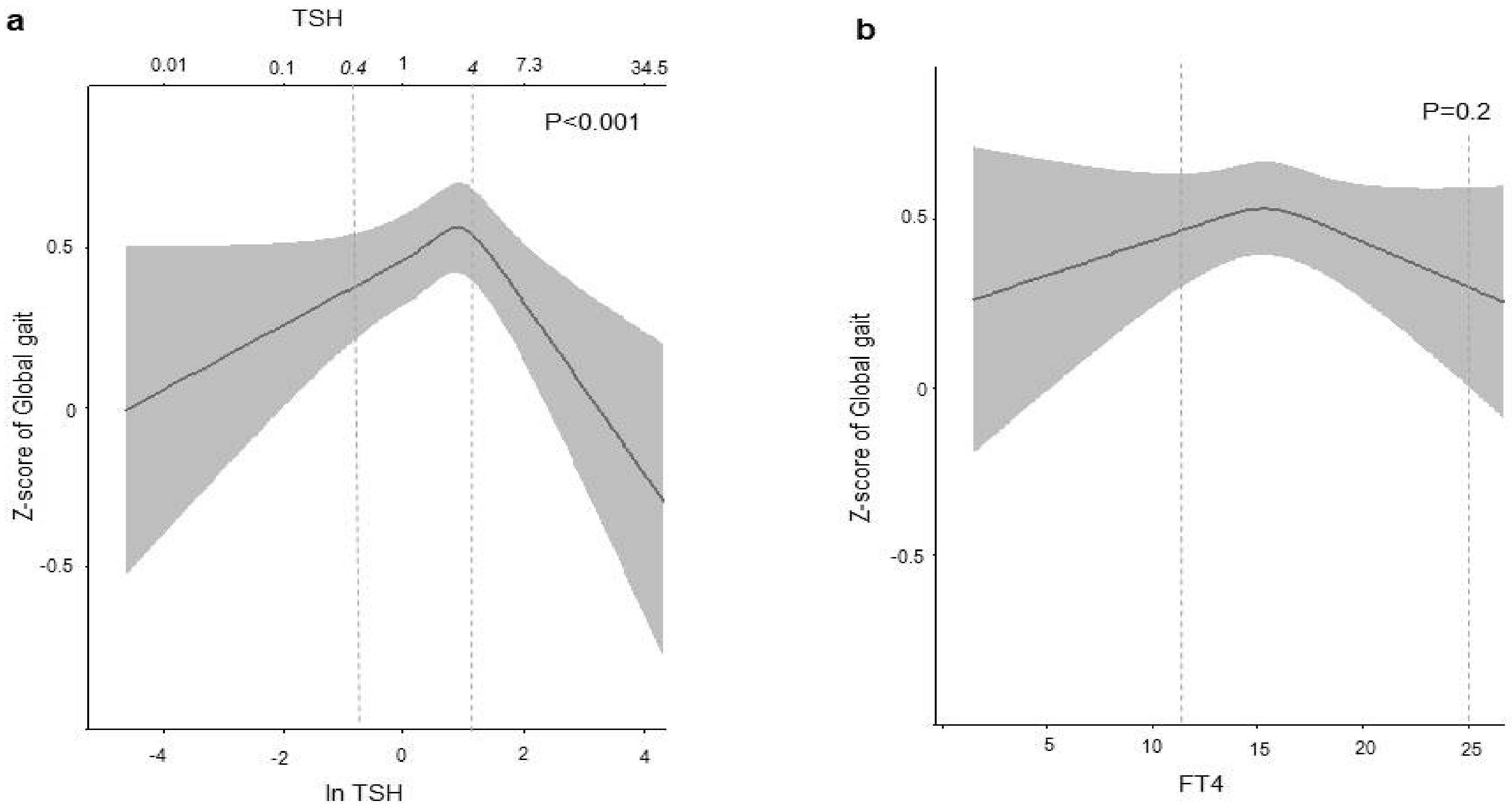

Supplement: Supplementary Information [file srep38912-s1.pdf]
